# Concurrent methicillin-resistant *Staphylococcus aureus* septicemia and pyomyositis in a patient with dengue hemorrhagic fever: a case report

**DOI:** 10.1186/s12879-018-3012-1

**Published:** 2018-02-27

**Authors:** N. D. B. Ehelepola, R. K. G. M. Rajapaksha, D. M. U. B. Dhanapala, T. D. K. Thennekoon, S. Ponnamperuma

**Affiliations:** The Teaching (General) Hospital – Kandy, Kandy, Sri Lanka

**Keywords:** Dengue, Dengue Hemorrhgic fever, Tropical pyomyositis, Methicillin-resistant *Staphylococcus aureus*, Septicemia, Sri Lanka

## Abstract

**Background:**

Concurrent presence of dengue hemorrhagic fever (DHF), tropical pyomyositis and septicemia due to methicillin-resistant *Staphylococcus aureus* (MRSA) in a previously healthy person has never been reported. These three conditions even individually are potentially fatal. “Here we describe a case of a patient contracting dengue and developing DHF along with concurrent pyomyositis likely to be due to MRSA, leading to MRSA septicemia with abscesses formed by MRSA”.

**Case presentation:**

A 44-year old previously healthy Sinhalese man presented on day 3 of the illness with fever, headache, arthralgia and myalgia and watery loose stools. His pulse rate was 76/min, blood pressure was 110/80 mmHg, while cardiovascular, respiratory and abdomen examination findings were unremarkable. The test for the dengue NS1 antigen was positive on the same day. We have diagnosed dengue and started managing him symptomatically as per the current national guidelines. The patient developed DHF with bilateral pleural effusion and ascitis. On the day 5 he developed severe myalgia, tenderness and non pitting edema of lower limbs especially in the thighs. His creatine kinase levels were high and an ultrasound scan confirmed myositis of both thighs. We suspected myositis due to dengue but investigated for possible simultaneous sepsis as well. On day 9 his blood culture became positive for MRSA. Considering the sensitivity of the bacteria intravenous vancomycin and ciprofloxacin was administered for 21 days. He developed a small abscess at the site of the first intravenous access and a large one above the ankle on the left. On day 12 the latter was drained and the pus culture yielded MRSA sensitive to the same antibiotics. The rapid test for dengue IgM was negative initially but later a positive MAC-ELISA test entrenched dengue infection. After improvement he was sent home on day 33 of the illness. He has developed two other abscesses in the proximity of the drained one and they were drained on day 57. The patient recovered.

**Conclusions:**

When dengue patients develop symptoms and signs of myositis, prompt investigations for pyomyositis and the treatment can save lives.

## Background

Dengue is a viral fever transmitted by *Aedes* mosquitoes. Out of some 96 million annual clinical dengue cases that are estimated to occur globally, the majority happens in developing tropical countries [1, 2]. Tropical pyomyositis otherwise known as tropical myositis, temperate myositis, pyogenic myositis, suppurative myositis, myositis purulenta tropica, infectious myositis, spontaneous bacterial myositis and epidemic abscess is a common primary infection of skeletal muscles usually caused by *Staphylococcus aureus* [3, 4].Large muscles of the thighs are the most commonly affected muscle group [4].The hallmark of the disease is myositis but abscess formations can also occur [3, 4].

There are only a few reports of dengue and MRSA co-infections despite the fact that both infections individually are common. Both dengue virus and *Staphylococcus aureus* individually are known to be capable of causing myositis [3, 5]. However, we found only one similar previous report: a case of dengue viral myositis complicated with a MRSA superinfection with a fatal outcome [5]. There is a report of a *Staphylococcus aureus* pneumonia and dengue co-infection with elevated creatine kinase levels and a case of multiple abscess formation by methicillin-sensitive *Staphylococcus aureus* in a dengue patient [6, 7]. Here we report a case of DHF in a previously healthy male complicated with simultaneous pyomyositis of thigh muscles giving rise to MRSA septicemia resulting in subcutaneous abcess formation by MRSA. Our patient has recovered. To the best our knowledge this is the first report of such a case.

## Case presentation

**Fig. 1:**
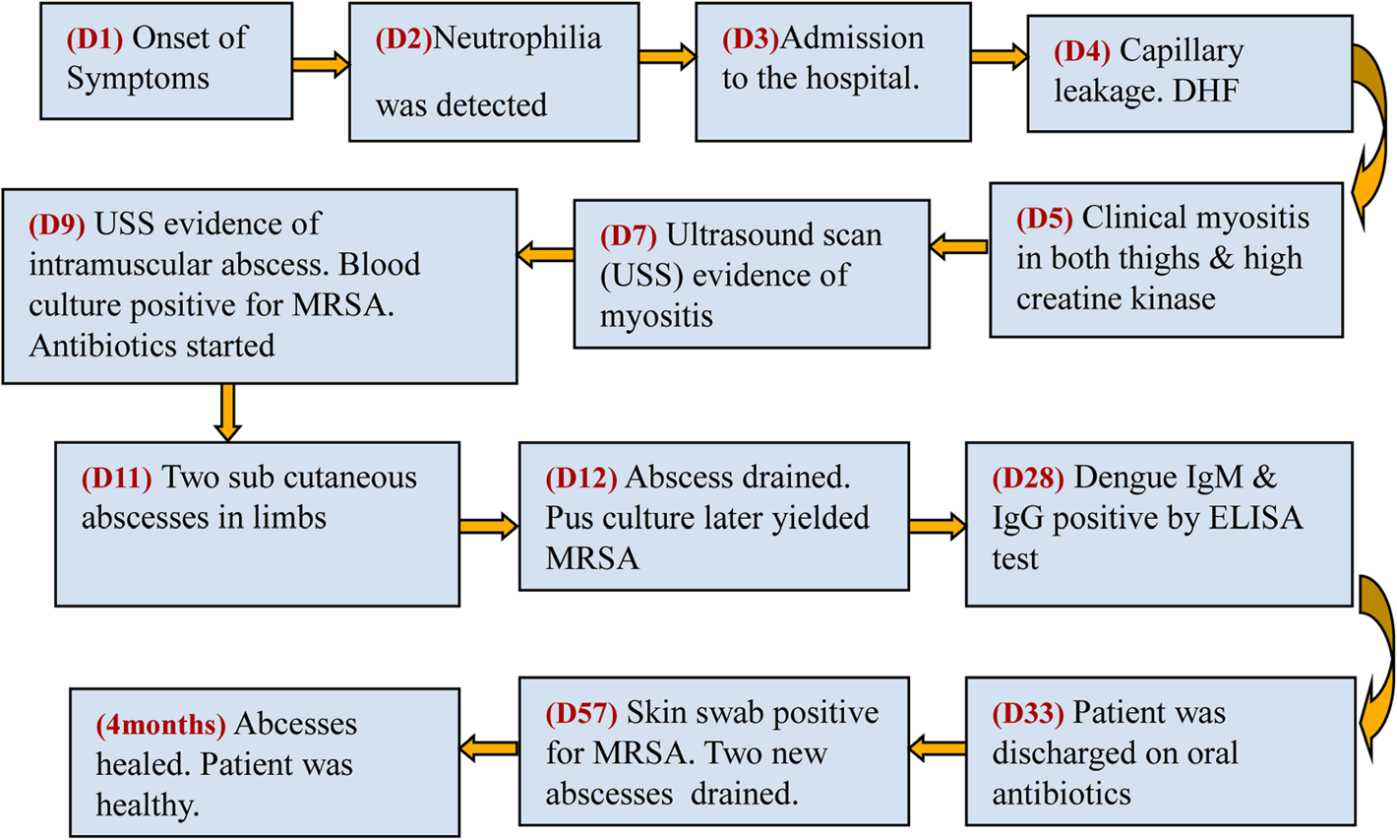
The timeline of salient events of his illness

A 44-year old previously healthy Sinhalese male presented on the third day (day 3) of the illness with high fever, headache, arthralgia and myalgia affecting all limbs and watery loose stools. Upon examination he was not dehydrated, there were no skin sepsis/rashes, pulse rate was 76/min, blood pressure was 110/80 mmHg and cardiovascular, respiratory and abdomen examination findings were unremarkable. The test for dengue NS1 antigen was positive on the same day (SD bioline NS1 kit, Standard Diagnostics Inc., Korea). His leukocyte count was 6.64 × 10^9^/l on the day 2 and 80.9% of that were neutrophils. We started managing him symptomatically for dengue as per the current (2012) national guidelines [8] which is similar to the World Health Organization’s 2009 guidelines for the management of clinical dengue [9]. The timeline below (Fig. 1) illustrates the sequence of salient events of his illness. We describe the events in detail following it.

On day 4 of the illness his platelet count dropped sharply from 190 × 10^9^/l to 47 × 10^9^/l, hematocrit showed > 10% rise from the day 2 value (baseline) for a brief period and he developed a mild cough. There was a moderate amount of free fluid in the peritoneal cavity but no pleural effusion in the ultrasound scan report. DHF was diagnosed. The patient had fever until day 5, after which it subsided from day 6 to day 8 of the illness. On day 5 there was a clinical bilateral pleural effusion as well, which was confirmed by ultrasound scan and by chest X-ray. On days 5–7 platelet count was 24-28 × 10^9^/l and then gradually rose. However he never had clinical bleeding manifestations.

On day 5 he complained of severe myalgia of both lower limbs especially in the thighs more on the left and there was severe tenderness and non pitting edema of the lower limbs. He could not walk during the days 5–10 of the illness. There was pain (without other signs of inflammation) in the intravenous cannula site in the right forearm and the cannula was removed. Creatine kinase was twice of the normal upper limit (372 IU/l, normally < 170 IU/l) and it had risen to a peak of 917 IU/l on day 8 and dropped to 235 IU/l on day 11. Urine myoglobin test was unavailable. We initially suspected the possibility of dengue myositis but considering the persistence of high neutrophil percentage, searched for evidence of sepsis as well. Ultrasound scan (USS) of both thighs done on day 7 confirmed inflammation of multiple muscles (heteregenous echogenicity) with subcutaneous tissue edema, there was fluid in between muscle compartments. Day 9 ultrasound scan demonstrated 1.4 × 5.3 mm fluid collection in left thigh muscles in addition, but without other evidence of abscess formation. Serial 12 lead electrocardiograms (ECG = EKG) and a 2D echocardiogram were done to exclude any possible myocardial involvement and the results were normal. On day 8 he developed a 3 mm size vesicles in the right forearm and a macular rash in both legs.

His C-reactive protein (CRP) level had risen from 37.4 mg/l (normally < 6 mg/l) on day 7 to 215.3 mg/l on day 9. On day 9 procalcitonin level was 13.5 ng/ml (> 2.0 ng/mL is highly suggestive of systemic bacterial infection). Leptospirosis is another locally prevalent infection that can give rise to a similar clinical picture [9]. IgM test for leptospirosis was negative on day 9. Blood culture became positive for MRSA on the same day. Intravenous (IV) vancomycin and ciprofloxacin was started considering antibiotic sensitivity and minimum inhibitory concentrations, and continued for the next 21 days. He had high fever again from day 9 to day 12 and there was another fever spike on day 15 and thereafter he was fever free until day 30 (while he was on intravenous antibiotics).

On day 9 we detected bilateral inguinal lymphadenopathy. On day 11 he developed a small < 4 mm diameter abscess at the site of the first intravenous access and a large 3 cm diameter one above the ankle on the left. On day 12 the latter was drained and the pus culture yielded MRSA sensitive to the same antibiotics. When repeated on day 23 (13 days after start of intravenous antibiotics) USS showed the inflammation of thigh muscles partially resolved and no fluid collections.

Blood cultures repeated on day 15 and day 22 were negative. IgM rapid test for dengue (SD bioline IgM/IgG kit, Standard Diagnostics Inc., Korea) gave negative results on days 15,21 and 22, in contrary to our expectations. However, on day 28 ELISA for dengue Ig M (MAC-ELISA) and Ig G test in 1:1280 dilution done at the national Medical Research Institute became positive confirming the diagnosis of DHF.

Day 17 USS of the chest showed pleural effusions had resolved.

His weight of 79 kg upon admission had reduced to 65 kg on day 29. He managed to regain 3 kg in the next 21 days. The antibody tests for HIV-1 and HIV-2 were negative.

Immediately after stopping IV antibiotics again on day 30 and 31 the patient developed small fever spikes. The repeated blood culture, CRP and procalcitonin levels and full (complete) blood count did not show evidence of infection and he was sent home on oral ciprofloxacin and co-trimoxazole on day 33. On day 50 he was reassessed. His repeated full (complete) blood counts, liver and renal function tests, CRP and repeated 2D echocardiography were all normal. The wound of the drained abscess was also partially healed. Swabs were taken for MRSA from the nostrils, throat, both axillae and groin regions. He came back with reports after a week. In the groin swab there was MRSA but not in others. A new one centimeter diameter abscess and a 2.5x1cm abscess was detected 2 cm proximal to the previous large abscess in the left lower limb and the abscesses were drained. 4% chlorhexidine body wash was prescribed to eliminate MRSA from his skin. Thereafter he has defaulted follow up. Four months after his first admission his abscesses were healed and he was apparently healthy.

## Discussion and conclusions

### Challenges faced in diagnosis and management

The patient’s clinical picture, pattern of drop of platelet count and rise again, drop of leukocyte count from 6.6 × 10^9^/l (normally 4–11 × 10^9^/l) on day 2, to 3.0 × 10^9^/l on day 3 and rise to 6.87 × 10^9^/l on day 4 and remaining above that thereafter, evidence of transient capillary leakage (bilateral pleural effusion and ascites) [9], and the fact that dengue is hyper-endemic in Sri Lanka and Kandy is one of the severely affected districts [10] all favor the diagnosis of DHF. He passed a larger urine volume than his daily fluid intake on days 8 and 9 indicating the recovery phase of DHF [8, 9]. Positive dengue NS1 antigen test on day 3 further supported the clinical diagnosis of DHF. Nonetheless the rapid test for dengue Ig M was negative thrice. The day before admission to our hospital (day 2) his neutrophil percentage was 80.9% and that remained high until day 15. Lymphocyte percentage remained low from day 2 to day 15 and the maximum value was only 28.9% on day 4. Those facts are not in favor of diagnosis of dengue [9]. Nevertheless, leukocyte counts and percentages can be well explained as a dengue and bacterial co-infection. There was a theoretical possibility that he had a MRSA septicemia alone (without dengue) as similar picture might occur due to MRSA exotoxemia causing capillary leakage and thrombocytopenia at the onset of sepsis [11, 12].

The initial clinical picture and positive NS1 antigen test, MAC-ELISA and Ig G ELISA tests confirmed the diagnosis of DHF [8, 9].

Dengue is hyperendemic and rising in Sri Lanka. Most Sri Lankan doctors known to us efficiently manage average DHF cases even without confirming the diagnosis serologically [10]. Nonetheless we believe ready availability of reliable tests (like MAC-ELISA) to confirm complicated cases of dengue like this case at major regional hospitals is a necessity.

The blood culture being positive for MRSA and pus culture of the lower limb abscess that appeared later yielding MRSA showing same antibiotic sensitivity pattern indicates MRSA septicemia subsequent to pyomyositis resulting in the abscess formation in the lower limb. The gold standard test for tropical pyomyosisits, biopsy of affected muscle and culture was not performed [4]. This was because we decided to administer IV antibiotics considering blood culture yielded MRSA and we were not going to depend upon this invasive procedure to alter the management. USS is also described as a good alternative to the biopsy and culture [4]. Serial USS of thigh muscles on both sides demonstrated inflammation and then resolving inflammation with treatment. The pattern of change of creatine kinase levels also supported the diagnosis of pyomyositis. Dengue viral myositis has heterogeneous presentations [13]. We do not know the degree of contribution to the inflammation of his muscles by dengue virus. The overall picture of this case favors the diagnosis of tropical pyomyositis caused by MRSA with DHF. Blood cultures are negative in > 90% cases of tropical pyomyositis in the tropics and in up to the half of cases a history of preceding blunt trauma or strenuous exercise of the affected muscles is there [3]. However, in our patient there was no such history and blood culture was positive. Positive blood culture enabled us to promptly treat him with the appropriate antibiotics. MRSA septicemia carry high mortality rates even with the best treatment and swift diagnosis and treatment are crucial for a good outcome [14]. Had this patient’s blood culture become negative, considering his clinical picture, high neutrophil percentage and high CRP and procalcitonin levels we still would have started a broad spectrum antibiotic like ceftriaxone or meropenem. However those would not have covered MRSA effectively and the patient’s outcome would have been worse. In such situations the availability of molecular diagnostic methods to supplement blood cultures [14] would be helpful for prompt and confident diagnosis of tropical pyomyositis due to MRSA. A great majority of DHF and pyomyositis cases occur in poorly resourced countries with limited laboratory and medical imaging facilities. Several tests of this patient were done in outside laboratories. The early transfer of patients to a hospital with facilities or starting an antibiotic that covers MRSA upon clinical diagnosis alone may save the lives of similar patients in such settings.

### When the MRSA infection occurred

Development of an abscess at the site of the first intravenous cannula indicated that as a potential site of MRSA entry. If that was the case MRSA that has been on his skin or from the hands of the nursing staff (who inserted the cannula) could have been the source. Two percent of the population carries MRSA [15]. A study from another Sri Lankan teaching hospital shows that > 10% of the nursing staff were at risk of transmitting MRSA [16]. Nonetheless considering the high granulocyte percentage even before admission to our hospital we believe bacterial infection (MRSA) had been going on even before he came to this hospital. The first stage of tropical pyomyositis (invasive stage) occurs over a period of a week [3, 4]. Critical phase of the DHF [8, 9] in this patient had occurred during the last days of the first stage of pyomyositis. Pyomyositis caused by community-acquired, methicillin-resistant *Staphylococcus aureus* (CA-MRSA) has risen in the recent past and this is likely to be such a case [4]. Concurrent bacterial infection has prolonged the hospital stay of our patient as well as of other reported cases [7, 17, 18].

Considering our experience and past reports [2, 17, 18], we believe secondary bacterial infections in dengue are common, especially in patients with severe forms of dengue. In fact during the 2017 epidemic we have witnessed many IV cannula site infections in dengue patients due to *Staphylococcus aureus* that usually responded to oral cloxacillin or flucloxacillin. However, a report from Singapore indicates concurrent bacteremia is uncommon in dengue patients in some countries [18]. Considering that up to 70% of patients with CA-MRSA soft tissue infections will experience recurrences over one year, even after successful initial treatment, we think the recurrence of his abscesses was not unusual and may have occurred in this case [19]. Nevertheless we have excluded HIV infection in him.

### Possible interactions between dengue, the immune system and MRSA

Information on the pathophysiological mechanisms of interactions between dengue and pathogenic bacteria are sparse [2]. We were unable to work out whether dengue infection or tropical pyomyositis occurred first in this patient.

Dengue patients are vulnerable to secondary bacterial infections due to leucopenia (especially neutropenia), impairment of antigen-presenting cells’ functions, reduction of the phagocytic and migratory capacities of macrophages, impairment of the interferon signaling pathway, impairment of the integrity of skin barrier against bacteria and other transient changes in the immune system caused by dengue virus [2, 8, 9, 17, 20, 21]. Increased capillary leakage of DHF is also speculated to favor bacterial infections [17]. Prior viral infections (especially arboviruses like dengue) are a known predisposing factor to tropical pyomyositis [3]. The exact pathophysiological mechanism responsible for that is unclear [3]. However, it is reasonable to speculate that the above mentioned mechanisms have contributed to the MRSA infection in this patient. All those factors reiterate the importance of precautions against healthcare associated infections (HAI) to prevent bacterial superinfections when managing dengue patients.

At the same time some existing bacterial infections make patients more prone to dengue infection [2].

MRSA was isolated from our patient’s groin skin even after a prolonged course of antibiotics. The dengue infection could have compromised his skin barrier and facilitated MRSA bacteremia [2] which gave rise to tropical pyomyositis.

## Conclusions

The diagnosis and management of concurrent tropical pyomyositis in dengue patients is challenging, especially in poorly resourced settings. We believe that it is worth looking for evidence of secondary bacterial infections in dengue patients if the fever is prolonged, the patient is very ill and if the neutrophil percentage does not decline during the course of the illness. Availability of tests for procalcitonin levels at hospitals may be helpful to detect bacterial co-infections in patients with viral fevers like dengue. If a dengue patient develops symptoms and signs of myositis, prompt investigations for pyomyositis and the treatment can save lives.

## Abbreviations

CA-MRSA: Community-acquired methicillin-resistant *Staphylococcus aureus*
CRP: C-reactive protein
DHF: Dengue hemorrhagic fever
ECG=EKG: Electrocardiograms
ELISA: Enzyme-linked immunosorbent assay
HAIs: Healthcare-associated infections
HIV: Human immunodeficiency virus
IV: Intravenous
MAC-ELISA: IgM antibody capture enzyme-linked immunosorbent assay
MRSA: Methicillin-resistant *Staphylococcus aureus*
USS: Ultrasound scan
